# Grassland songbird abundance is influenced more strongly by individual types of disturbances than cumulative disturbances associated with natural gas extraction

**DOI:** 10.1371/journal.pone.0283224

**Published:** 2023-03-17

**Authors:** Stephen K. Davis, Holly J. Kalyn Bogard, David Anthony Kirk, Lauren Moretto, R. Mark Brigham

**Affiliations:** 1 Environment and Climate Change Canada, Canadian Wildlife Service, Regina, Saskatchewan, Canada; 2 Department of Biology, University of Regina, Regina, Saskatchewan, Canada; 3 Aquila Conservation & Environment Consulting, Ottawa, Ontario, Canada; Feroze Gandhi Degree College, INDIA

## Abstract

Grassland birds have undergone widespread global population declines due to loss and degradation of native grasslands. Activities associated with non-renewable energy derived from oil and natural gas extraction have substantially increased on grasslands. The cumulative disturbance generated by natural gas development creates a network of non-linear (e.g., bare ground and exotic plant species) and linear (e.g., roads, trails, pipelines) features that may degrade habitat quality for grassland species. We quantified grassland songbird abundance in two areas of southwestern Saskatchewan, Canada, to determine whether variation in abundance 1) depended on the type and amount of disturbance at two spatial extents, and 2) was more affected by the cumulative impacts of natural gas development than any single type of disturbance. We found that specific types of disturbances impacted the abundance of most species to varying degrees. The cover of different types of linear disturbance had the strongest effect on the most species. Natural gas disturbance within 450 m of point counts was more influential than disturbance within 200 m for nearly all species in both areas. Only Savannah sparrow (*Passerculus sandwichensis*) abundance was most strongly influenced by the cumulative amount of disturbance with abundance decreasing with increased disturbance. Overall, we detected few consistent patterns among species, or within species between our two study areas. Our results indicated that the impact of natural gas infrastructure can extend beyond the local influences associated with well sites and that relatively small amounts of disturbance (<2%) may impact grassland songbird abundance. We recommend that researchers use caution when studying well-density effects or combining individual types of disturbance without understanding the separate effects each type of disturbance has on the species or community of interest. Not doing so may lead to investing resources into management practices that do not have the greatest possible benefit for grassland songbirds.

## Introduction

The loss and degradation of grasslands through intensification of agricultural practices has led to widespread global declines in grassland bird populations [1–3]. The decline in grassland bird populations has continued despite a plateau, or even a decrease, in the global amount of grassland being converted to annual crops over the past 60 years [4, 5]. These population declines may be related, in part, to a substantial increase in industrial development over time, such as oil and gas extraction [6, 7]. Petroleum development has the potential to destroy and degrade the quality of remaining grasslands and therefore impact grassland bird populations. Copeland et al. [8] predicted that approximately 15% of grasslands in western North America could be impacted by oil and gas development.

Most research on the biological effects of oil and gas development in grasslands has focused on well sites, specifically the type, density, and proximity of wells [9–12]. This research focus likely relates, in part, to the information requirements imposed by regulators regarding permitting, establishing mitigation guidelines, and reporting on drilling activity [13]. Negative impacts from the construction of well sites have been demonstrated for a number of taxonomic groups, including arthropods [14], amphibians [15], mammals [10, 16], and birds [9, 17].

Construction of well pads and other infrastructure destroys local grassland cover and may impact the broader landscape, having long-lasting effects on ecosystems [14, 18, 19]. For example, bare ground cover is greater and vegetation is shorter near natural gas wells, due in part to well construction activities and livestock congregating around well sites [11, 19, 20]. These changes in vegetation structure likely alter bird species composition and abundance in the landscape surrounding the well. Bird species that require relatively sparse and short vegetation may be more abundant closer to wells or in areas with higher well densities, whereas bird species requiring taller and denser vegetation may be less abundant near wells.

Despite these predictions about bird community composition, the demonstrated effects of well density and proximity on grassland songbirds have been relatively weak and inconsistent among and within species and regions. For example, Kalyn Bogard and Davis [11] found little evidence of bird abundance being strongly correlated with proximity to gas wells after accounting for vegetation effects. Additionally, the authors found that the abundance of two species associated with relatively short and sparse vegetation [horned lark (*Eremophila alpestris*) and chestnut-collared longspur (*Calcarius ornatus*)] increased with well density at northern sites, but not at southern sites. In contrast, Rodgers and Koper [20] found that horned lark abundance decreased with well density in southeastern Alberta. These inconsistent results within and among studies, may be due to variation in the type and age of infrastructure, vegetation, and/or the type and extent of anthropogenic disturbance present on the landscape. Furthermore, the cumulative effects of multiple anthropogenic disturbances (e.g., roads, pipelines, noise, and pollution) may have a stronger influence on grassland songbird demography than gas well proximity or density alone [21].

Cumulative effects are temporally and spatially additive effects of similar individual actions, or the interactive effects of different multiple actions that alter the structure or function of ecosystems [22]. Each single type of anthropogenic disturbance may not have a large effect on species’ habitat quality on its own, but the cumulative effects of multiple anthropogenic disturbances may be profound [22]. In the case of natural gas wells, the cumulative disturbance generated by development creates a network of non-linear (e.g., bare ground and exotic plant species) and linear (e.g., roads, trails, and pipelines) features. Aside from increased bare ground cover, well pads often have greater amounts of exotic plant species, such as crested wheatgrass (*Agropyron cristatum*), either planted as part of a restoration program or accidentally seeded by humans via vehicles and equipment, or livestock [18, 19]. Crested wheatgrass alters vegetation structure and ecosystem function [23, 24], which may lower grassland songbird density and reproductive success [25, 26]. Natural gas infrastructure can also provide perch sites for brown-headed cowbirds (*Molothrus ater*) which parasitize the nests of other songbirds, leading to reduced reproductive success of some songbird hosts near infrastructure [27]. Lands with natural gas development also contain linear features, such as roads and trails, that are used for routine maintenance and monitoring of well sites, and dredged pipeline networks that link wells to a central compressor station. Linear features may impact grassland songbirds by increasing the amount of edge vegetation in the landscape and by serving as conduits for predators [28–30] and invasive plant species which degrade habitat quality [31, 32].

We used an information theoretic approach [33] to test the following hypotheses at two local spatial extents (200 m and 450 m radius buffer around point counts): 1) The extent to which grassland songbird abundance varies with natural gas disturbance depends on the type and amount of disturbance (Disturbance-type hypothesis). We expected that the variation in abundance would be best explained by specific types of individual disturbance variables, with some being more influential than others. 2) Grassland songbird abundance is more affected by the cumulative impacts of natural gas development than any single type of disturbance alone (Cumulative effects hypothesis). We expected that grassland songbird abundance would be most affected by the cumulative amount of disturbance within a given area. Our study builds upon previous work by Kalyn Bogard and Davis [11] which focused on the effect of local disturbance factors influencing grassland songbird abundance. Our study also contributes to the body of knowledge regarding the effect of natural gas development on prairie wildlife at multiple spatial scales.

## Methods

### Study area

We conducted our study on five Agriculture and Agri-Food Canada (AAFC) community pastures in southwestern Saskatchewan in 2008 and 2009 [Battle Creek 287.0 km^2^, Govenlock 280.3 km^2^, Nashlyn 249.7 km^2^, Big Stick 121.1 km^2^ and Bitter Lake 199.9 km^2^; see Kalyn Bogard and Davis [11] for a map and detailed description of the study area and pastures]. Natural gas development was the only type of industrial activity present on the five pastures during the study. We selected these large pastures to avoid the possibility of confounding effects of patch size [34, 35] and landscape configuration [36] on grassland bird abundance.

We divided our study region into a northern study area comprising Bitter Lake and Big Stick pastures and a southern study area that included Govenlock, Nashlyn, and Battle Creek pastures. The northern and southern study areas were approximately 120 km apart. The northern pastures were characterized by sandy loam—sand soils, and southern pastures primarily by solonetzic soils [37]. Mid-height grasses (*Stipa* spp., *Koeleria macrantha*, *Pascopyrum smithii* and *Elymus macrourus*) were predominant in the former area, whereas short grass species (*Bouteloua gracilis* and *Carex* spp.) were dominant in the latter area. As expected, bird species favoring taller and denser vegetation were more abundant in the northern pastures, whereas species associated with sparser and shorter vegetation were more abundant in the south [11]. In addition, the density of natural gas wells and the legacy of their development differed between the two areas. In the northern study area, installations began in the 1950s and averaged nine gas wells/1.6 km^2^ at the time of our study. Drilling for natural gas began in the 1990s in the south, and averaged 0.8 wells/1.6 km^2^ at the time of our study. All parcels were grazed by cattle and stocking rates were 0.03 to 0.06 animal unit months/ha [see Kalyn Bogard and Davis [11] for more details].

### Avian surveys

All protocols were approved by the President’s Committee on Animal Care at the University of Regina and the Canadian Wildlife Service Animal Care Committee (Protocol 09–02). We used legal sections (2.59 km^2^ or 259 ha) of land (hereafter ‘sections’) based on the Dominion Land Survey system [38] and summed the number of gas wells within each section using a Geographic Information System (GIS). Each well pad had a single well, there were no multi-well pads on any of our sites. We stratified sections into one of four well-density categories (wells/section): high (10), medium (5–9), low (1–4), and zero wells. Within each randomly-selected section, we also randomly-selected two point-count locations within 0–100, 101–200 m and > 200 m of randomly-selected gas wells to ensure that bird survey locations represented a range in the type and extent of disturbances from natural-gas development [see Kalyn Bogard and Davis [11] for details]. Point-count centers were at least 400 m apart to avoid recording the same individual birds more than once due to overlapping detection distances.

We quantified songbird abundance using 5 min, 100 m radius point-counts conducted from the third week in May to the end of June in 2008 and 2009. Surveys were conducted from 30 min before sunrise to four hours thereafter in good weather conditions (winds < 20 km/hour and no precipitation). All birds seen or heard within and outside the 100 m radius circle were recorded by observers following a one minute ‘settling period’ after arrival at the point-count location [39]. We attempted to use distance sampling [40] to account for the probability that the detection of individuals decreases with increasing distance from the observer. However, we recorded few detections close to the observer and thus violated the assumption that the detection probability at distance 0 = 1.0 [11]. Therefore, we did not adjust our abundance estimates using a distance function. Instead, we used removal sampling [41] to account for the probability that birds elicited an aural or visual cue during the count period. We achieved this by dividing the count duration into three sequential time intervals of 100 sec each and recording detections of new birds within each time interval. Because their detection was influenced by well density and proximity, we adjusted the raw counts for horned lark and Savannah sparrow (*Passerculus sandwichensis*) based on the modeled detection function and rounded them to the nearest integer [42]. For all other species, we used raw counts.

### Natural-gas development disturbance

We conducted extensive ground-truthing within the pastures after bird surveys had been completed. Locations of all trails, roads, pipelines, well sites, bare ground, exotic vegetation patches, exclusion fencing, and compressor stations were recorded with a Global Positioning System (GPS) unit and converted to line and polygon features. We calculated an average width for each linear feature based on 25 randomly-selected roads, trails, and pipelines within the study area. We then converted lines to polygon features by buffering each linear feature using its average width in a GIS. Non-linear features (exclusion fencing, bare ground, and exotic vegetation cover) were delineated by walking the perimeter of each disturbance feature and logging waypoints with a handheld GPS unit. We outlined continuous areas of bare ground and exotic vegetation that were at least 1m in diameter. We did not delineate areas that were naturally devoid of vegetation because of the underlying soil (e.g., alkali, gravel, etc). Waypoints were then imported into a GIS and used to create polygons for each of the features. We created 200 m and 450 m buffers around each point-count location and calculated the proportion of each linear and non-linear feature within the two buffers. We used these proportional values as explanatory variables in all analyses.

### Statistical analyses

We investigated the effects of natural gas development on grassland bird abundance at two different spatial extents using generalized linear mixed models (GLMM) or generalized linear models (GLM), depending on model convergence outcomes. We created GLMMs and GLMs using the GLIMMIX and GENMOD procedure in SAS v9.4 [43], respectively. We used pasture as a random effect for the GLMM analyses and modeled both GLMMs and GLMs with species’ counts as the response variable with a negative binomial distribution and a log link. We built separate models for the northern and southern study areas because of regional differences in well densities, underlying soils, age of gas wells, and plant and avian communities (see above). We created models using both 200 m and 450 m radius buffers to assess the degree to which the effect of natural gas disturbance on grassland birds was influenced by local spatial extents. We used a 200 m buffer as our smallest extent in an attempt to encompass the territories of the birds counted within the point count area. We limited our largest extent to a 450 m radius buffer around point-count centers because larger extents included different land cover types unrelated to natural gas development (e.g., cropland, tame forage, and large water bodies) and for logistical purposes (e.g. landowner permission and ability to conduct detailed ground-truthing). Although limiting the size of our landscapes likely reduced the influence of different land cover types on the birds in our study, we recognize that they could still exert some influence.

We identified 10 disturbance variables composed of linear and non-linear features that might be expected to influence grassland songbird abundance: the number of gas wells, the length of exclusion fencing surrounding new gas wells, and the cover of gravel roads (municipal and well-access roads), trails (dirt and grass), pipelines, exotic vegetation, bare soil, and overall disturbance (S1 Table). We first examined whether the effect of any of these variables on abundance depended on the year of the study to inform whether we could pool the data across years in our modelling. We compared a linear model with an additive effect of year (e.g., wells + year) and a model with an interactive effect of year (e.g., wells + year + wells × year) using the Akaike Information Criterion (AIC; [33]). If there was strong support for an interactive effect (interactive model > 2 AIC units smaller than the linear model and 85% confidence intervals excluded 0), we examined the strength and direction of the relationship for both years. In all cases where the interactive model was supported, it was because the effect of the natural gas variable was stronger in one of the years and not because there was a statistically significant positive effect in one year and a negative effect in the other. Therefore, we pooled the two years of data for all analyses.

We created a suite of 11 *a priori* models at two spatial extents for both the northern and southern study areas and compared them using AIC: 1) number of wells, 2) pipeline cover, 3) exotic vegetation cover, 4) bare ground cover, 5) length of exclusion fencing, 6) exotics + bare ground (representing non-linear disturbance), 7) dirt trail cover + grass trail cover (representing trails), 8) municipal grid road cover + well road cover (representing roads), 9) linear features (non-overlapping cover of roads, trails and pipelines combined), 10) cumulative disturbance (total cover of area disturbed by any form of gas development and municipal grid roads), and 11) an intercept-only model (null). For each species, we considered the model with the lowest AIC value to be the top-performing model of the models we examined. We identified competing models as those that were within 2 AIC units of the top model with the same or fewer number of parameters. Furthermore, we considered models having AIC values at least 2 units smaller than the null model with parameters having 85% CI excluding zero to also be influential [44].

## Results

We recorded a total of 11 songbird species across both the northern and southern study areas and over both years of sampling, including horned lark, Sprague’s pipit (*Anthus spragueii*), chestnut-collared longspur, thick-billed longspur (*Rhyncophanes mccownii*), grasshopper sparrow (*Ammodramus savannarum*), clay-colored sparrow (*Spizella pallida*), vesper sparrow (*Pooecetes gramineus*), Baird’s sparrow (*Centronyx bairdii*), Savannah sparrow, and western meadowlark (*Sturnella neglecta*). We had sufficient data to evaluate abundance models in both the northern and southern regions for horned lark, Sprague’s pipit, chestnut-collared longspur, and Baird’s sparrow. However, we had to confine our analyses to the northern region only for grasshopper sparrow, clay-colored sparrow, vesper sparrow, Savannah sparrow, and western meadowlark, and to the southern region for thick-billed longspur and Brewer’s sparrow because of insufficient data.

**Fig 1:**
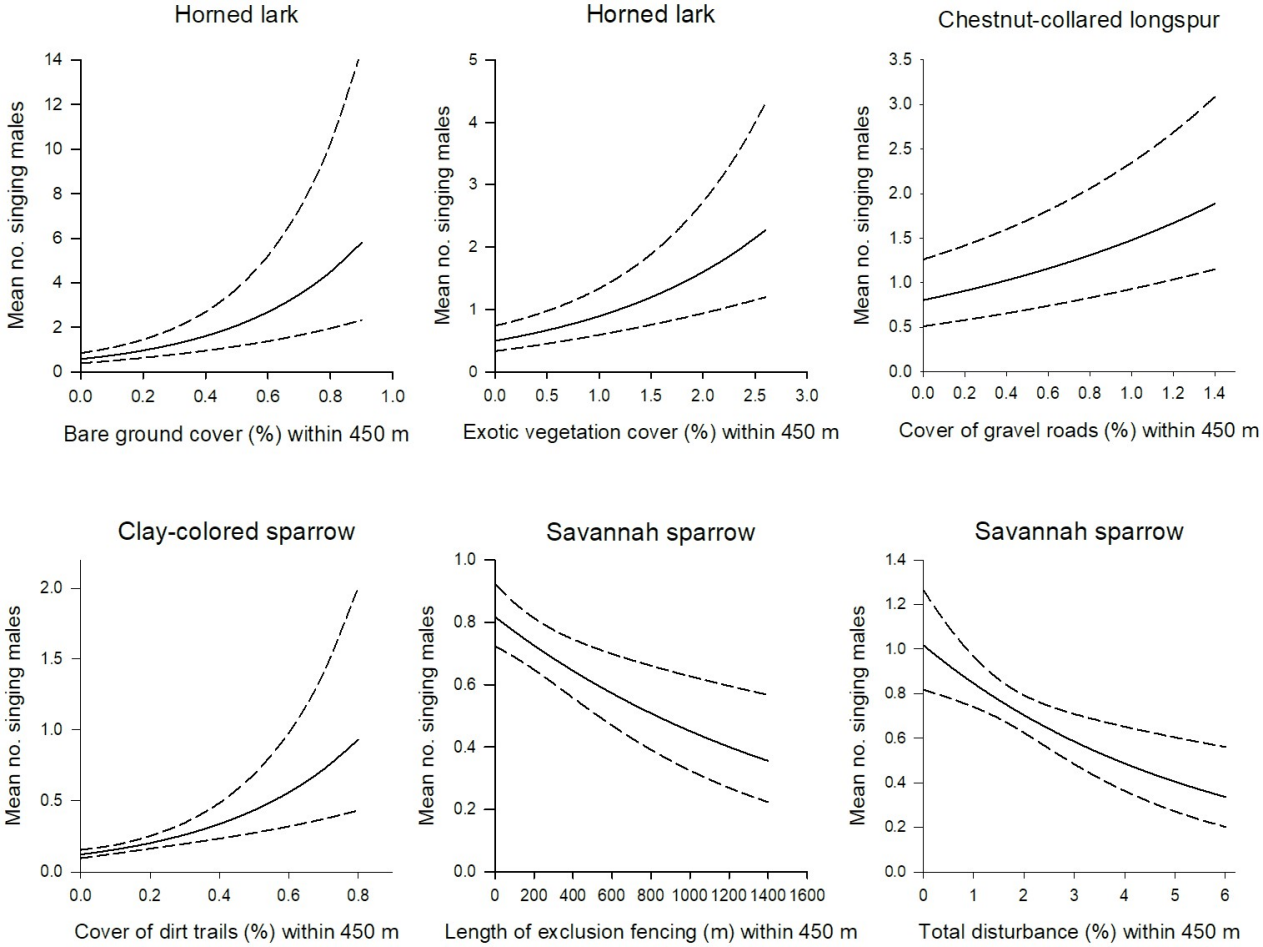
Grassland songbird abundance in the northern study area varies as a function of different types of natural gas disturbance within a 450 m radius buffer. Dashed lines are 85% confidence limits.

Abundance of most species was influenced by specific types of natural gas disturbances with some individual disturbances being more influential than others. Cumulative disturbance was a top or competing model only for Savannah sparrow (Table 1). Savannah sparrow abundance declined with increased amount of cumulative disturbance and exclusion fencing within a 450 m radius buffer (Fig 1). Overall, songbird abundance was best explained by natural gas disturbance variables assessed at the 450 m radius extent (Table 1). Only chestnut-collared longspur and thick-billed longspur abundance in the south was best explained by variables at the 200 m extent. However, in both cases, the top models were within 2 AIC units of the null model suggesting limited support (Table 1). Similarly, we found little support for natural gas disturbance strongly affecting the abundance of horned lark in the southern area and Sprague’s pipit and vesper sparrow in the northern area as the top models were within 2 AIC units of the null model (Table 1).

**Table 1:** Top (lowest AIC values) and competing (within 2 AIC units of top model with as many or fewer parameters) models explaining variation in abundance of grassland songbirds as a function of natural gas disturbance at the 200 m and 450 m radius extent in the northern and southern study areas.

Species	Model	K	*DAIC*	*Wi*
Horned lark				
North	Bare450 (**2.57 ± 0.67**), Exotics450 (**0.58 ± 0.16**)	5	0.0	0.90
	Null	3	22.9	0.00
South	Exotics450 (-**0.38 ± 0.19**)	4	0.0	0.19
	Null	3	1.8	0.08
Sprague’s pipit				
North	Bare450 (-**1.77 ± 1.01**)	3	0.0	0.16
	Exotics200 (-**0.68 ± 0.43**)	3	0.9	0.10
	Disturb200 (-**0.34 ± 0.21**)	3	1.3	0.08
	Wells450 (-**0.07 ± 0.04**),	3	1.4	0.08
	Null	2	1.9	0.06
South	Wells450 (-**1.11 ± 0.31**)	3	0.0	0.84
	Null	2	16.7	0.00
Chestnut-collared longspur				
North	Grid450 (-0.14 ± 0.25), Gravel450 (**0.61 ± 0.14**)	5	0.0	0.92
	Null	3	14.4	0.00
South	Grid200 (**0.95 ± 0.50**), Gravel200 (-**2.56 ± 1.72**)	4	0.0	0.19
	Null	2	1.8	0.08
Thick-billed longspur				
South	Bare200 (-**8.18 ± 5.35**)	4	0.0	0.18
	Wells200 (-**0.32 ± 0.18**)	4	0.4	0.14
	Null	3	1.6	0.08
Grasshopper sparrow				
North	Grid450 (-**1.53 ± 0.69**), Gravel450 (-0.01 ± 0.25)	5	0.0	0.19
	Wells200 (-**0.21 ± 0.13**)	4	1.5	0.09
	Dirt450 (**0.89 ± 0.54**), Grass450 (-0.76 ± 0.71)	5	1.6	0.09
	Wells450 (-**0.10 ± 0.07**)	4	1.8	0.08
	Null	3	2.5	0.05
Clay-colored sparrow				
North	Dirt450 (**2.54 ± 0.76**), Grass450 (-1.62 ± 1.19)	4	0.0	0.55
	Null	2	12.2	0.00
Brewer’s sparrow				
South	Grid450 (0.84 ± 0.66), Gravel450 (**2.63 ± 0.68**)	5	0.0	0.91
	Null	3	10.4	0.00
Vesper sparrow				
North	Wells450 (**-0.17 ± 0.09**)	3	0.0	0.22
	Exotics450 (**-0.53 ± 0.35**)	3	1.5	0.11
	Null	2	1.9	0.09
Baird’s sparrow				
North	Pipeline450 (**0.37 ± 0.12**)	3	0.0	0.46
	Wells450 (-**0.08 ± 0.03**)	3	1.5	0.21
	Null	2	8.1	0.01
South	Grid450 (-**1.63 ± 0.60**), Gravel450 (**-0.97 ± 0.59**)	5	0.0	0.78
	Null	3	9.1	0.01
Savannah sparrow				
North	Fence450 (-**0.001 ± 0.0002**)	3	0.0	0.21
	Disturb450 **(-0.18 ± 0.08**)	3	0.3	0.19
	Null	2	3.7	0.03
Western meadowlark				
North	Pipeline200 (**2.72 ± 0.93**)	3	0.0	0.48
	Null	2	6.3	0.02

See S1 Table for a description of explanatory variables and S1 Appendix for detailed results for all models considered. Parameter estimates and associated standard errors in bold indicate 85% confidence intervals that exclude zero. K is the number of model parameters, *D*AIC represents the number of AIC units from the top model in the 200-m and 450-m extent suite of models combined, and *Wi* is the AIC weight of evidence that the model is the best of those considered.

**Fig 2:**
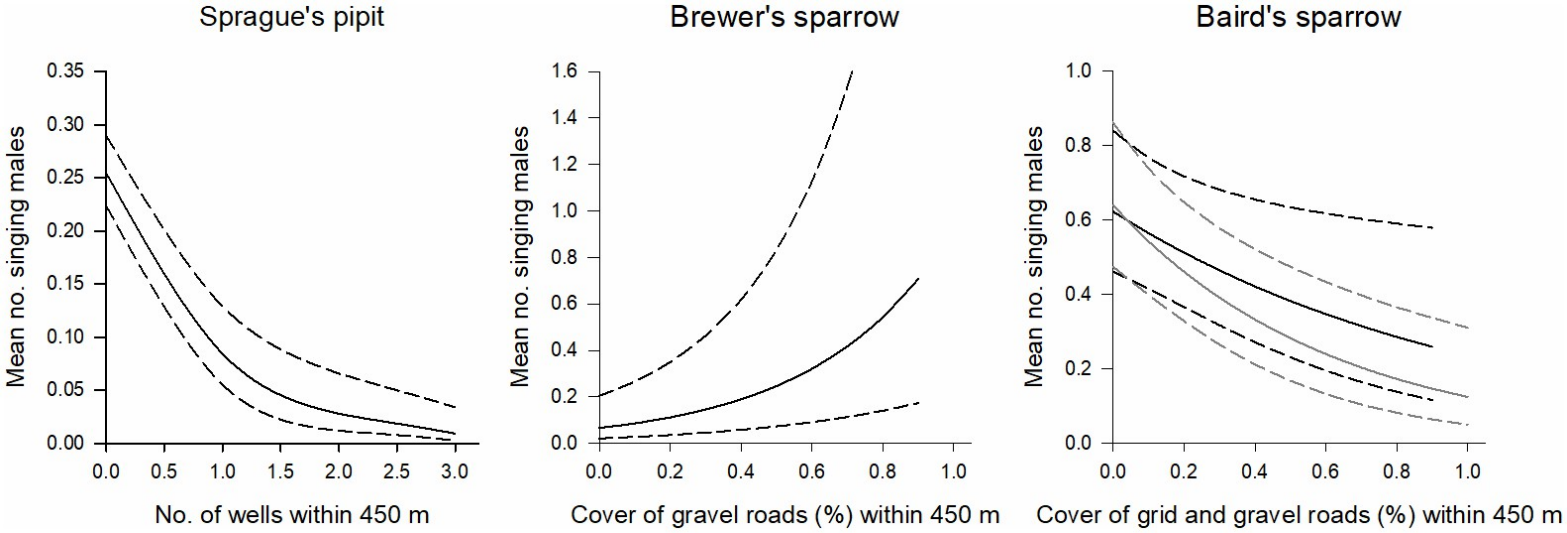
Grassland songbird abundance in the southern study area varies as a function of the number of gas wells and the cover of roads within a 450 m radius buffer. Dashed lines are 85% confidence limits and gray lines represent grid roads and black lines represent gravel roads.

Of the 10 natural gas disturbance models we considered, we found strong support for a single model explaining variation in songbird abundance for horned lark, chestnut-collared longspur, and clay-colored sparrow in the northern area, and Sprague’s pipit, Brewer’s sparrow, and Baird’s sparrow in the southern area. AIC weights, ranged from 0.55 to 0.92 and no other model was within 2 AIC units of any of the top models (Table 1). Horned lark abundance in the north increased with the cover of bare ground and exotic species within a 450 m radius buffer and chestnut-collared longspur abundance increased with the cover of gravel roads (Fig 1). Clay-colored sparrow abundance increased with the cover of dirt trails (Fig 1). In the southern area, Sprague’s pipit abundance declined as the number of wells within 450 m increased, whereas Baird’s sparrow abundance declined with the amount of roads (Fig 2). In contrast, Brewer’s sparrow abundance increased with the amount of gravel roads within 450 m (Fig 2).

**Fig 3:**
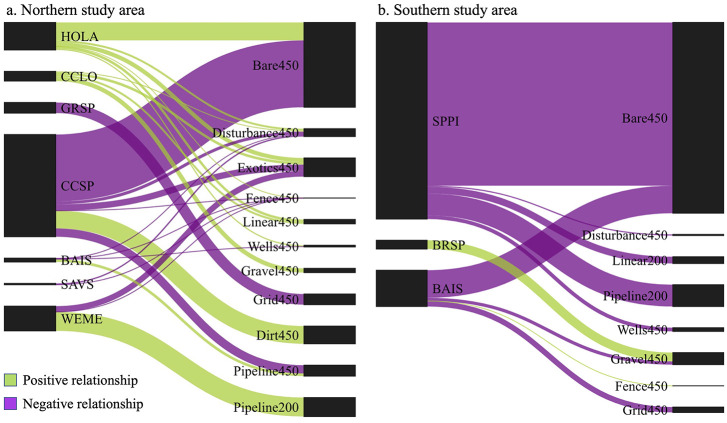
Direction and strength of relationships between grassland songbird abundance and influential natural gas disturbance variables. Shown are standardized parameters with 85% confidence limits that exclude 0. Size of the lines represents the relative strength of the relationship with thicker lines having larger standardized parameter estimates. HOLA = horned lark, SPPI = Sprague’s pipit, CCLO = chestnut-collared longspur, GRSP = grasshopper sparrow, CCSP = clay-colored sparrow, BRSP = Brewer’s sparrow, BAIS = Baird’s sparrow, SAVS = Savannah sparrow, and WEME = western meadowlark. Thick-billed longspur (TBLO) and vesper sparrow (VESP) model results are not shown because top models were within 2AIC units of the null model. See S1 Table for explanation of explanatory variables.

Examination of other models that were at least 2 AIC units smaller than the null model with parameter estimates having 85% confidence limits excluding zero revealed a variety of natural gas disturbances that may also influence songbird abundance in our region (Fig 3, S1 Appendix). For some species, natural gas disturbances had both negative and positive effects on abundance (clay-colored sparrow, Baird’s sparrow and western meadowlark) whereas other species exhibited either negative (Sprague’s pipit and Savannah sparrow) or positive effects (horned lark and chestnut-collared longspur) (Fig 3).

## Discussion

The ‘Disturbance-type hypothesis’ (the extent to which grassland songbird abundance varies with natural gas disturbance depends on the type and amount of disturbance) received substantial support. Not only was the abundance of most species influenced by specific types of disturbances but some disturbances were clearly more influential than others. Interestingly, some disturbances had a positive effect on abundance. This suggests that some features of natural gas infrastructure may provide some benefit to some species (e.g., horned lark and chestnut-collared longspur), but information on reproductive success and survival are needed to determine if lands under natural gas development act as an ecological trap or not [45]. Overall, we detected few consistent patterns among or within species between the two study areas, likely due in part to regional differences in well densities, age of gas wells, and plant communities. However, abundance of most species was consistently best explained by landscape variables at the 450 m radius extent versus the 200 m radius extent.

The ‘Cumulative effects hypothesis’ (grassland songbird abundance is more affected by the cumulative impacts of natural gas development than any single type of disturbance alone) received limited support as Savannah sparrow was the only species whose abundance was most strongly influenced by the cumulative amount of disturbance on the landscape. Cumulative disturbance may not have been the strongest predictor of abundance because the effects of individual disturbances differ interspecifically. Consequently, the result of pooling disturbance features into one variable for some species may obscure the effects of individual features.

Linear features (e.g., roads, trails, and pipelines) were an important type of impactful disturbance, with the cover of roads and trails affecting the abundance of five species and pipeline cover influencing the abundance of four species. We expected the cover of municipal grid roads to have the greatest detrimental impact on abundance because they have a larger footprint and are typically associated with increased traffic and noise compared to natural gas access roads and trails [46, 47]. However, only the abundance of grasshopper and Baird’s sparrow in the south were negatively affected by municipal grid road cover. Furthermore, the magnitude of the decline for Baird’s sparrow was similar for both municipal grid roads and gravel access roads. In contrast to Baird’s sparrow, the abundance of chestnut-collared longspur in the north and Brewer’s sparrows in the south increased with the cover of gravel roads. This is consistent with Nenninger and Koper [17] who showed that Baird’s sparrow abundance was greater away from roads and chestnut-collared longspur abundance was greatest closer to roads. In contrast, Daniel and Koper [21] found the abundance of chestnut-collared longspur to be greater away from roads. It is difficult to reliably account for differences among studies. Inconsistencies among studies are likely a function of the types of disturbances examined (e.g., paved roads versus municipal grid roads versus gravel well-access roads), combining different types of disturbances into broad categories (e.g., roads, linear features), different scales, methodologies and analyses, and conducting studies in different years and regions or under different environmental conditions and development histories.

Habitat loss due to roads often accounts for the largest proportion of land-use change in areas undergoing energy development [21, 48]. Roads and trails are thought to negatively influence grassland bird demography due to traffic, anthropogenic noise, and edge effects. Such features increase predator and brood-parasite densities and/or predator foraging efficiency [29, 49] leading to reduced density, reproductive success, and survival [50–52]. It is unlikely that traffic and anthropogenic noise played a major role in influencing the abundance of species along roads and trails in our study given the low traffic volumes (see also [53]). This is a consequence of the sparse human population in the region and the infrequent maintenance associated with gas wells [19]. In addition, others have reported little evidence for anthropogenic noise and traffic impacts in mixed-grass prairie rangelands [12, 17].

The extent to which natural gas development promotes the growth of edge vegetation, which in turn lowers demographic rates of grassland songbirds, is difficult to assess. Edge effects tend to be more prominent in landscapes where the structure of the edge vegetation contrasts markedly with that of the interior vegetation [54]. In contrast to grassland-woodland ecotones, the structure of edge vegetation created by roads, trails, and pipelines in mixed-grass rangelands may not differ enough to meaningfully alter predator species composition, movement, density, or foraging efficiency [55, 56]. Furthermore, grassland songbird predators are extremely diverse and opportunistic [57, 58], and some primary predators of songbirds are more common in the interior of grasslands rather than along the edges [59, 60]. Conversely, other studies examining edge effects in landscapes inundated with oil and gas development have found evidence for abundance, reproductive success, and clutch size to increase farther from roads, but the relationships are highly variable within and among species and studies [20, 21, 25, 51, 61]. Edge effects in our study may arise more from changes in vegetation structure, with vegetation being shorter and sparser near roads and trails [20]. This may explain, in part, the patterns we observed with Baird’s, grasshopper, and Savannah sparrows, species that were more abundant in landscapes with less road cover and are associated with relatively tall and dense vegetation. Similarly, chestnut-collared longspur is a species that was more abundant in landscapes with increased road cover and is associated with relatively short and sparse vegetation. In addition, more perch sites may be available near roads in the form of fence lines and shrubs as a result of disturbance associated with construction of roads and dirt trails. Consequently, species strongly associated with shrubs such as Brewer’s and clay-colored sparrows [62, 63] may be more abundant in areas with increased road and trail cover.

Construction of gas wells and associated infrastructure alters the structure of the plant community through the removal of topsoil, soil compaction, and in pastures, the interactive effects of cattle grazing [19]. Consequently, species associated with relatively short and sparse vegetation (e.g., horned lark and chestnut-collared longspur) were more abundant in landscapes with increased bare ground cover, whereas those associated with taller and denser vegetation (e.g., Sprague’s pipit and Baird’s sparrow) were less abundant. In addition, direct seeding of exotic species in past mitigation practices and invasion by introduced species also change the composition and structure of the vegetation community [19, 64]. These changes influenced the abundance of six species, but the strength and direction of the relationships varied.

The influence of exclusion fencing on abundance also varied in terms of the strength and direction of the relationships. Exclusion fencing may attract some species by providing perches for singing, but discourage others because they avoid relatively tall anthropogenic structures or because fences also provide perches for predators and brown-headed cowbirds. Fences may also affect abundance, not because of the infrastructure itself, but rather because of the vegetation within and around the fenced area. Fences are erected to allow the vegetation to recover by excluding cattle. Therefore, depending on when the fencing was erected, vegetation structure in and around the fenced area may range from sparse and short with relatively high cover of bare ground or it may resemble a state closer to its pre-drilling condition. These scenarios are potential explanations for the variation in strength and direction of the relationships we found.

Investigations into the impact of natural gas development on songbirds have often concentrated on local-scale effects such as vegetation structure and proximity to infrastructure [11, 20, 51, 65]. However, factors considered across larger spatial extents may be better predictors of occurrence or abundance than smaller scales [66, 67], making it important to consider how industrial development across larger spatial extents influences bird populations [21, 68]. The spatial extent used to assess natural gas development effects in mixed-grass prairie has often been based on legal land subdivisions at the section (2.59 km^2^ or 259 ha) level [11, 21, 51, 69], presumably because well density at that scale is a common metric used by the oil and gas industry and regulators [69]. It is unclear what spatial extents are most relevant for assessing the effect of oil and gas development on grassland songbirds. Some studies have not detected a significant relationship between songbird occurrence/abundance and well density at the section level [20, 69] while others have [11, 21]. Hamilton et al. [69] found the amount of disturbance (pipeline, well pad, and trails combined) within a 100 m radius buffer influenced the occurrence of two of three species examined. Mutter et al. [68] found little support for local scale effects of wells and roads, but did find that well and road density within a 2 km buffer significantly influenced the abundance of two of the three songbird species studied. We found that the amount of disturbance within a 450 m radius buffer was consistently more influential than at the 200 m radius extent. Although the primary reason we used a 450 m radius buffer was because it was the largest extent we could accommodate without overlapping different landscapes and land uses, it does have practical applications. Land-use cover at a similar-sized landscape (400 m radius buffer versus 800 m and 1600 m radius) was found to be the best predictor of abundance and reproductive success for most grassland songbirds in other regions of the northern mixed-grass prairie [26, 70]. Furthermore, the area of the circular buffer closely represents the same area as a legal quarter-section, which is a typical unit of land managed for pasture and natural gas development [38]. Clearly, further research is required to determine the most appropriate spatial extents for assessing the impact of natural gas development on grassland songbird demography.

## Conclusions

Understanding the effect of natural gas development on grassland species is essential for developing science-based mitigation and management prescriptions. We have assumed that changes in vegetation structure, habitat fragmentation, and avoidance of natural gas infrastructure are the primary factors influencing grassland songbirds in areas inundated by natural gas development. However, animals may also be affected by environmental factors related to the natural gas life cycle, such as pollutants in the water, soil and air [71, 72], although such research for grassland songbirds is lacking.

Our results indicate that grassland songbirds are affected by specific types of disturbance with some types of disturbance being more influential than others. Surprisingly, well density, a common factor examined in wildlife-petroleum studies [11, 20, 51], had little influence on grassland songbird abundance overall compared to other sources of disturbances. Consequently, research and management focused only on well sites will likely yield suboptimal conservation measures for grassland songbirds. Furthermore, our results indicate that researchers should be cautious assuming that well density is an appropriate proxy for the overall amount of disturbance in an area, particularly if multi-well pads are common. Although well density was positively correlated with cumulative disturbance in our study (Pearson r = 0.58–0.73; S1 Appendix), well density models had significantly different AIC values than cumulative disturbance models for a number of species, confirming that the two variables were not redundant (S1 Appendix). Researchers should also be wary when combining individual types of disturbance into commonly used categories such as roads, trails, linear features, and cumulative disturbance without understanding the individual effects each type of disturbance has on the species or community of interest. Not considering individual effects of disturbances may result in pooling disturbance types that affect a given species in different ways, ultimately leading to misdirected management strategies and misinformed policy. A number of studies have investigated local effects of energy development on wildlife [11, 12, 20]. Our results indicate that the amount of disturbances, even those with a small physical footprint (e.g., <2% of a 450 ha area), may impact grassland songbird abundance. However, we acknowledge that the birds in our study could be influenced more strongly, and even in the opposite direction, at larger spatial scales [73]. Further research on the effects of different types of natural gas disturbances at varying spatial scales is required to understand the drivers of spatial and temporal variation in the response of grassland species to energy development.

## Supporting information

S1 Table Description of explanatory variables used in models predicting relationships between grassland songbird abundance and natural gas development disturbance features. (DOCX)

S1 Appendix Pearson correlation coefficients (r) of disturbance variables examined at both sites (north and south) and spatial extents (200m and 450 m radius) along with the complete set of models used to evaluate the influence of natural gas disturbance on grassland songbird abundance. Also included with the models are their Akaike Information Criterion (AIC) ranks, AIC weights, parameter estimates and 85% confidence limits. See S1 Table for description of explanatory variables. HOLA = horned lark, SPPI = Sprague’s pipit, CCLO = chestnut-collared longspur, TBLO = Thick-billed longspur, GRSP = grasshopper sparrow, CCSP = clay-colored sparrow, BRSP = Brewer’s sparrow, VESP = vesper sparrow, BAIS = Baird’s sparrow, SAVS = Savannah sparrow, and WEME = western meadowlark.
(XLSX)
