# Supplementary material for: Grassland songbird abundance is influenced more strongly by individual types of disturbances than cumulative disturbances associated with natural gas extraction
Source: PLoS One. 2023 Mar 17;18(3):e0283224. doi: 10.1371/journal.pone.0283224 (PMC10022753; doi:10.1371/journal.pone.0283224)
Supplement: S1 Table — (DOCX) [file pone.0283224.s001.docx]

**S1 Table**. **Description of explanatory variables used in models predicting relationships between grassland songbird abundance and natural gas development disturbance features.**

| Explanatory variable (short form) | Description |
| --- | --- |
| Natural gas wells (Wells200, Wells450) | Number of gas wells within 200 m and 450 m of the point-count center. |
| Exclusion fencing (Fence200, Fence450) | Chain-link fencing (height=2m) used to exclude grazing around recently drilled gas wells. Total length (m) of fencing within 200 m and 450 m of the point-count center. |
| Gravel road  (Gravel200, Gravel450) | The cover (%) of graded roads with gravel surface within 200 m and 450 m of the point-count center. Roads are used primarily by energy companies to access gas wells and compressor stations. Roads may or may not have an associated ditch. |
| Municipal grid road  (Grid200, Grid450) | The cover (%) of graded roads with a gravel surface within 200 m and 450 m of the point-count center. Roads have a raised bed and straddled by ditches and are maintained by the rural municipality for public use. |
| Dirt trail  (Dirt200, Dirt450) | Cover (%) of a graded trail within 200 m and 450 m of the point-count center. The trail is not raised or covered with any other substrate and is typically used to access gas wells, gain entry into pastures, and double as fire breaks. |
| Grass trail  (Grass200, Grass450) | Cover (%) of ungraded, 2-track trails within 200 m and 450 m of the point-count center. Trails are used to access well sites and pasture interiors. |
| Pipeline  (Pipeline200, Pipeline450) | Cover (%) of pipelines within 200 m and 450 m of the point-count center. Pipelines are used to carry natural gas to compressor stations and storage facilities. Pipelines are underground but surface features differ from surrounding grassland. Disturbed ground above pipelines typically characterized by less vegetative cover and intentionally seeded with exotic vegetation (typically crested wheatgrass) or invaded by exotic vegetation. |
| Linear features  (Linear200, Linear450) | Total amount of non-overlapping linear disturbance combined (roads, trails and pipelines) within 200 m and 450 m of the point-count center. |
| Exotic vegetation  (Exotics200, Exotics450) | Cover (%) of exotic vegetation within 200 m and 450 m of the point-count center. Typically comprises crested wheatgrass, but does not include exotic vegetation planted for livestock forage. |
| Bare soil  (Bare200, Bare450) | Cover (%) of bare soil within 200 m and 450 m of the point-count center. Includes patches of bare ground created from removal and compaction of top soil (e.g., construction of well pad). |
| Total disturbance  (Disturb200, Disturb450) | Total amount of non-overlapping disturbance combined within 200 m and 450 m of the point-count center. |
